# The characteristics and effectiveness of pregnancy yoga interventions: a systematic review and meta-analysis

**DOI:** 10.1186/s12884-022-04474-9

**Published:** 2022-03-25

**Authors:** Lisa Corrigan, Patrick Moran, Niamh McGrath, Jessica Eustace-Cook, Deirdre Daly

**Affiliations:** 1 School of Nursing & Midwifery, Trinity College Dublin, Dublin, Ireland; 2 School of Public Health, University College Dublin, Dublin, Ireland; 3 Library of Trinity College Dublin, Dublin, Ireland

**Keywords:** Pregnancy yoga, Systematic review, meta-analysis, FITT principle

## Abstract

**Background:**

Yoga is a popular mind-body medicine frequently recommended to pregnant women. Gaps remain in our understanding of the core components of effective pregnancy yoga programmes. This systematic review and meta-analysis examined the characteristics and effectiveness of pregnancy yoga interventions, incorporating the FITT (frequency, intensity, time/duration and type) principle of exercise prescription.

**Methods:**

Nine electronic databases were searched: MEDLINE, PsycINFO, EMBASE, CINAHL, WHOLiS, AMED, ScieLo, ASSIA and Web of Science. Randomised control trials and quasi-experimental studies examining pregnancy yoga interventions were eligible. Covidence was used to screen titles, abstracts, and full-text articles. Outcomes of interest were stress, anxiety, depression, quality of life, labour duration, pain management in labour and mode of birth. The Cochrane Collaboration’s Risk of Bias Assessment tool was used to assess methodological quality of studies and GRADE criteria (GRADEpro) evaluated quality of the evidence. Meta-analysis was performed using RevMan 5.3.

**Results:**

Of 862 citations retrieved, 31 studies met inclusion criteria. Twenty-nine studies with 2217 pregnant women were included for meta-analysis. Pregnancy yoga interventions reduced anxiety (SMD: -0.91; 95% CI: − 1.49 to − 0.33; *p* = 0.002), depression (SMD: -0.47; 95% CI: − 0.9 to − 0.04, *P* = 0.03) and perceived stress (SMD: -1.03; 95% CI: − 1.55 to − 0.52; *p* < 0.001). Yoga interventions also reduced duration of labour (MD = − 117.75; 95% CI − 153.80 to − 81.71, *p* < 0.001) and, increased odds of normal vaginal birth (OR 2.58; 95% CI 1.46–4.56, *p* < 0.001) and tolerance for pain. The quality of evidence (GRADE criteria) was low to very low for all outcomes. Twelve or more yoga sessions delivered weekly/bi-weekly had a statistically significant impact on mode of birth, while 12 or more yoga sessions of long duration (> 60 min) had a statistically significant impact on perceived stress.

**Conclusion:**

The evidence highlights positive effects of pregnancy yoga on anxiety, depression, perceived stress, mode of birth and duration of labour.

Systematic review registration: PROSPERO, CRD42019119916. Registered on 11th January 2019.

**Supplementary Information:**

The online version contains supplementary material available at 10.1186/s12884-022-04474-9.

## Background

Pregnancy is characterised by significant physiological, social and emotional changes which can impact on maternal and fetal health and well-being across multiple domains [1, 2]. There is comprehensive evidence that anxiety, depression, and stress in pregnancy are risk factors for adverse maternal and fetal outcomes ranging from preterm birth and low birth weight to adverse neurodevelopmental outcomes in infants and children [3, 4]. The well-being of the mother is therefore critical for optimal pregnancy and child outcomes. Pregnant women should be provided with support, tools, resources, and appropriate types and amounts of physical activity during pregnancy to reduce the risk of complications and promote optimal pregnancy and birth outcomes [5].

Yoga is a mind-body-spirit practice combining physical postures, relaxation, and breathing techniques [2, 6]. It has been adapted for the pregnant body and is a common form of physical activity used by pregnant women and recommended by healthcare professionals [2, 7–9]. Evidence suggests that yoga during pregnancy is safe, feasible and acceptable to pregnant women and may be more beneficial than walking and standard prenatal exercises for both physical and mental health [5, 10, 11]. It is also thought to provide pregnant women with the opportunity to foster well-being and develop a connection with their baby [5, 12]. Two randomised control trials (RCTs) of pregnancy yoga report that it lowers levels of pain, stress, anxiety and depression [13, 14]. A third systematic review of yoga for pregnant women concluded that overall, pregnancy yoga RCTs resulted in improvements in stress levels, quality of life (QoL), autonomic nervous system functioning and labour parameters such as comfort, pain and duration [2].

However, other systematic reviews identified wide variation in pregnancy yoga intervention characteristics, the degree of supervision of the yoga interventions, the sample population and outcomes measured, and recommended further exploration of these factors in future trials [15]. Two recent meta-analyses demonstrated that yoga was an effective complementary treatment to manage prenatal depression and improve mode of birth outcomes [16, 17]. Both studies also identified limitations; women recruited to included studies commenced yoga practice at different gestational ages and yoga interventions varied in terms of frequency, type and intensity across trials. While the body of evidence supporting the positive impact of pregnancy yoga on pregnancy and birth outcomes is growing, there is a need to pool evidence from studies to accurately measure treatment effect and explore the mechanisms by which yoga contributes to reported benefits [2, 15]. This should include analysis of the characteristics of the pregnancy yoga interventions in order to design programmes that can offer optimal benefit.

The success of physical activity (PA) interventions is said to depend on four factors: how often you exercise, how hard you exercise, how long you exercise, and the types of exercise you choose. These factors make up the frequency, intensity, time/duration and type (FITT) principle and are frequently used to describe PA intervention characteristics [18]. The objective of this systematic review was to examine the published evidence on pregnancy yoga, describe the characteristics of each intervention using the FITT principle of exercise prescription and assess the overall effects of pregnancy yoga on a range of identified outcomes [18].

## Materials and methods

### Protocol

This systematic review and meta-analysis were planned and conducted in accordance with Preferred Reporting Items for Systematic Reviews and Meta-Analyses (PRISMA) guidelines (Additional file 1), the PROSPERO registered (CRD42019119916) and HRBopen published protocols and the recommendations of the Cochrane Collaboration [19–21].

### Search strategy

The following electronic databases were searched from their inception up to November 2021:

MEDLINE (EBSCO), CINAHL (EBSCO), PsycINFO (EBSCO), Embase (Embase.com), AMED (EBSCO), WHOLiS, Web of Science (Clarivate), ScieLo (Clarivate) and ASSIA (Proquest). The search strategy was constructed around search terms for “pregnancy” and “yoga” and adapted for each database, as necessary. No language or date restrictions were included. Each concept was searched individually compiling terms using the OR Boolean operator and then the two concepts were combined using the AND operator. PICOS (population or problem, intervention, comparator, outcomes, study design) framework was established and guided the selection process. Additional file 2 contains this framework and the search terms and search strategy for Embase.com. Reference lists of included studies and relevant reviews were screened to ensure all suitable studies were identified. Grey Literature search of Proquest dissertations and theses, LENUS, RIAN, Google Scholar, and relevant journal conference supplements was also conducted. Only peer-reviewed published studies were included. The initial search was run on 22nd January 2019, updated on 22nd May 2020 and again on 5th November 2021.

### Selection criteria

#### Participants

Both normal healthy and high-risk pregnant women of any gestation, age, ethnicity and country of residence.

#### Intervention

Studies where yoga was the primary intervention delivered to a sample of pregnant women. Multimodal interventions delivering yoga in conjunction with other treatments for pregnant women were excluded.

#### Comparison

Pregnant women receiving usual care or any active treatment other than yoga.

#### Outcomes

Primary outcomes of interest were stress, anxiety, depression and quality of life. Secondary outcomes were birth outcomes of labour duration, pain management in labour and mode of birth. Included studies had to assess at least one primary or secondary outcome measured using validated self-report or clinician-rated questionnaires, measures or scales or by clinical diagnosis or medical chart review.

#### Study design

Any primary study that investigated a pregnancy yoga intervention within a RCT or quasi-experimental study with a control before and after design was considered for inclusion. Case control studies, crossover trials and cross-sectional studies were excluded.

### Information retrieval and data extraction

Search results were exported to EndNote X9 (Clarivate) and duplicate records removed (LC and JEC) [22]. Records were exported (JEC) to Covidence (Veritas Health Innovation), a web-based software platform designed to support citation screening and collaboration amongst multiple authors [23].

Author pairs (LC and DD, LC and PM, LC and NMcG) independently screened abstracts and the full text of potentially eligible studies according to inclusion/exclusion criteria, with third-party arbitration available if needed. Reasons for excluding studies at full-text review were recorded. The PRISMA flow diagram was used to show the overall process of study selection and summarise the inclusion and exclusion of studies at each stage of the review [19].

A standardised data extraction tool (Additional file 3) was developed specifically for this review based on recommendations provided in the Cochrane Handbook of Systematic Reviews of Interventions (LC) [21]. Author pairs (LC and NMcG; LC and PM) independently extracted data on study design and methods, sociodemographic characteristics, inclusion and exclusion criteria, study setting, details of experimental intervention and comparison intervention, duration of follow-up and outcomes studied, and extent of effectiveness. Discrepancies were discussed with another review author (DD) until consensus was reached. If necessary, study authors were contacted up to three times via email at fortnightly to provide further details. Data were entered into the RevMan 5.3 software and checked for accuracy (LC) [24].

### Quality assessment and assessment of confidence in the review findings

The Cochrane Collaboration’s tool for assessing risk of bias was used to evaluate the quality of the studies [25]. Risk of bias assessment was undertaken by author pairs (LC and NMcG; LC and PM) independently. Discrepancies were resolved by discussion with a fourth reviewer (DD), if required. Where reported information was unclear or where data were missing three attempts were made to contact the primary authors for clarification.

Quality of the evidence was evaluated using the Grades of Recommendation, Assessment, Development and Evaluation (GRADE) approach [26]. GRADEpro GDT software was used to import data from RevMan 5.3 and create the ‘Summary of findings’ Table [27]. Two review authors (LC and PM) graded the quality of the evidence for each outcome. Lack of double blinding alone was not downgraded due to difficulties blinding participants and yoga instructors. Downgrading was based on risk of bias only if a lack of blinding was accompanied by additional high risk of bias (e.g., selection bias and incomplete outcome reporting). It should be noted that the GRADE tool was developed for use in RCTs where double blinding was possible [26]. A summary of intervention effects and a measure of quality according to the GRADE approach was determined for seven outcomes; maternal stress, maternal anxiety, maternal depression, maternal QoL, duration of labour, pain management and mode of birth.

Results from included studies are presented as odds ratios (OR) with 95% confidence intervals (CI) for dichotomous outcomes. The mean difference (MD) was used for continuous data where outcomes were measured in the same way between trials, and the standardised mean difference (SMD) was used where outcomes were measured differently. The outcome measures from the individual trials were combined through meta-analysis where possible (clinical comparability of populations, interventions, outcomes and time of assessment between trials) using a random-effects model. According to the Cochrane Handbook for Systematic Reviews of Interventions a random-effects model offers the most conservative estimate of effect when between-study variations exist [25]. Data from studies that were too dissimilar to combine in a meta-analysis were described narratively in the text. Statistical heterogeneity was assessed in each meta-analysis using the T^2^, I^2^ and chi square statistics [25].

Subgroup analysis applying the FITT principle of exercise prescription to stratify results by frequency, intensity, time/duration and type, where appropriate, was conducted. Any statistically significant subgroup effect was reported using the *p*-value from the test for subgroup differences. The I^2^ statistic was used to measure the magnitude of heterogeneity in each sub-group and categorised according the Cochrane Handbook for Systematic Reviews of Interventions as follows: heterogeneity might not be important (I^2^ value 0–40%), moderate heterogeneity (I^2^ value 30–60%), substantial heterogeneity (I^2^ value 50–90%) or considerable heterogeneity (I^2^ value 75–100%) [25].

Sensitivity analysis to compare including and excluding RCTs at high risk of bias was conducted for stress (perceived), depression, duration of labour and mode of birth based on identification of studies with notably higher risk of bias.

## Results

### Results of the search

**Fig. 1:**
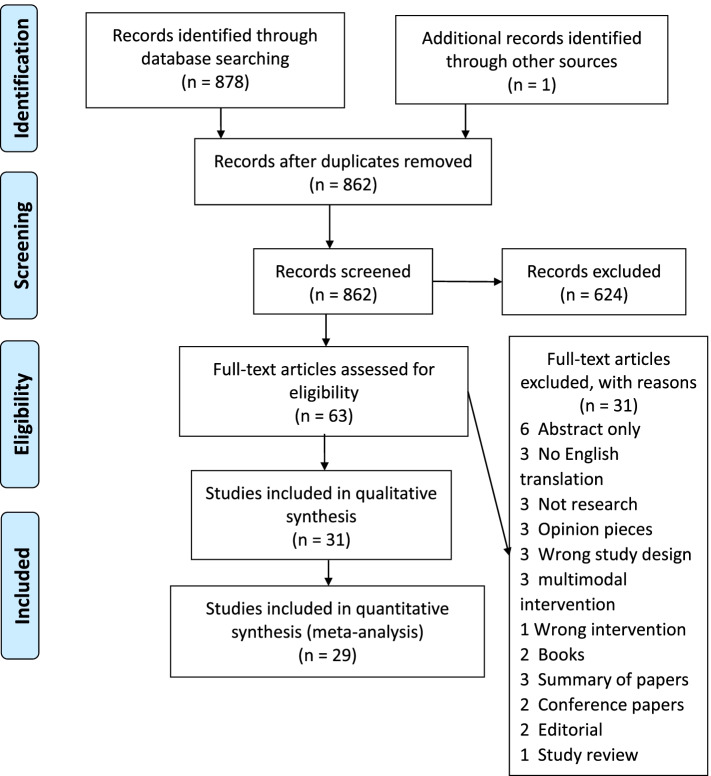
PRISMA flow diagram [19]

In total 862 records were identified and 62 retained for full-text screening (Fig. 1). Thirty-one studies including 2413 pregnant women were included in the review and study sample sizes ranged from 20 to 335. Data from 29 studies including 2217 pregnant women were suitable for and included in the meta-analysis. Two studies were not included because data could not be disaggregated for meta-analysis and they are reported narratively instead.

### Study characteristics

The characteristics of the included studies according to the FITT principle of exercise prescription are described (Table 1). Thirteen of the included studies originated from India [13, 28–39] eight from the USA [8, 11, 40–45], three from Iran [46–48], two each from China [49, 50] and Indonesia [51, 52] and one each from Japan, Thailand and the UK [53–55]. Twenty-five of the studies were RCTs, three were non-randomised control trials and three were a true-experimental post-test only control group design. Twenty studies were conducted with normal healthy pregnant women [8, 13, 29–31, 34–36, 38, 43, 46–55], two with multi-factor high-risk pregnant women [28, 32], six with pregnant women with depression or symptoms of depression [11, 39–42, 44], one with pregnant women with gestational diabetes [33], one with pregnant women with mild hypertension [37] and one with high-risk pregnant women on bedrest [45]. The gestational age at recruitment across studies ranged from 12 to 36 weeks. Control groups included routine antenatal care, usual activity, standard antenatal exercise walking 30 min twice daily, health education, social support, mom-baby wellness workshops, and parenting education sessions.

**Table 1:** Characteristics of studies according to FITT principle

Study ID	Country	Study type	Sample size	Gestation (weeks)	Intervention	Control	Outcome of interest	Main results	Frequency	Intensity	Timing	Type
Babbar et al. 2016	USA	RCT uncomplicated pregnancy	46 (23/23)	28–36 weeks	Yoga session	PowerPoint presentation	Mode of birth	NVB 65% yoga and 61% control	One time	One	60 mins	Poses, breathing, shavasana
Balaji et al. 2017	India	RCT gestational diabetes	151 (75/76)	24 weeks	Yoga sessions	Routine treatment	Mode of birth	NVB 84% yoga 26% control	Daily	3 months taught if practiced daily availability of 91 sessions	60 mins	Loosening exercises, postures, deep relaxation technique, pranayama
Bershadsky et al. 2014	USA	Non-randomised control trial normal pregnancy	50 (38/12)	12–19 weeks	Yoga sessions	Usual activity	Depression Physiological stress	Cortisol levels lower post yoga and fewer depressive symptoms in yoga group	No information	No information taught sessions	90 mins	Hatha yoga
Bhartia et al. 2019	India	RCT low risk pregnant women	78 (38/40)	18–20 weeks	Yoga therapy	Routine physical activity	Perceived stress Mode of birth	Perceived stress reduced 31.75% in yoga group and increased 6.60% in control (*p* < 0.001). NVB 92% yoga and 90% control	Tri-weekly	36 sessions – 12 taught & 24 self-practice	50 mins	Loosening exercises, breathing, postures, deep relaxation
Bolanthakodi et al. 2018	India	RCT normal pregnancy	150 (75/75)	30 weeks	Yoga therapy	Standard antenatal care	Mode of birth Pain management	More NVB in yoga group (*p* < 0.037), duration of labour was significantly shorter (*p* < 0.001) Significant reduction in intravenous analgesic in yoga group (*p* < 0.045) and tolerance of pain was higher as shown by NPIS (*p* < 0.001) and PBOS scores (*p* < 0.0001)	Bi-weekly for 4 sessions and weekly for three sessions and self-practice tri-weekly	7 taught sessions & availability of 24–36 self- practice sessions	30 mins	Integrated approach to yoga therapy (IAYT)
Chen et al. 2017	China	RCT healthy pregnant women	94 (48/46)	16 weeks	Yoga sessions	Routine prenatal care	Physiological stress	Prenatal yoga significantly reduced cortisol (p < 0.001)	Bi-weekly	40 taught sessions	70 mins	Postures, deep breathing, guided imagery, deep relaxation
Chuntharapat et al. 2008	Thailand	RCT normal pregnancy	74 (37/37)	26–28 weeks	Yoga sessions	Routine nursing care	Pain management Duration of labour	No differences between groups for pethidine usage. Shorter duration of labour in yoga group	Bi-weekly taught and tri-weekly self-practice	6 taught sessions & 30–36 available self-practice sessions	60 mins	Education, postures, chanting om, breath awareness, dhyana, yoga nidra
Davis et al. 2015	USA	RCT symptoms anxiety/depression	46 (23/23)	28 weeks	Yoga sessions	TAU	Depression Anxiety	Prenatal yoga was associated with reductions in symptoms of anxiety and depression	Weekly	8 taught sessions and self-practice DVD	75 mins	Ashtanga Vinyasa system of yoga modified for pregnancy
Deshpande et al. 2013	India	RCT high-risk pregnancies	68 (30/38)	12 weeks	Yoga therapy	Standard antenatal care/prenatal stretching exercises	Perceived stress	RMANOVA showed a significant decrease (*P* = 0.02) in the PSS scores of the yoga group compared to the control group	No information	16 weeks	No information	No information
Field et al. 2012	USA	RCT depression	56 (28/28)	20 weeks	Yoga postures	Standard prenatal care	Depression Anxiety	Decreased depression scores (F = 82.40,*p* < 0.001) and decreased anxiety scores (F = 26.23, *p* < 0.001) in the yoga group	Bi-weekly	24 taught sessions	20 mins	Postures
Field et al. 2013	USA	RCT depression	92 (46/46)	22 weeks	Yoga postures	Social support	Depression Anxiety Physiological stress	Reduced anxiety and depression in both groups with no significant group difference and reduced cortisol pre/post yoga and pre/post social support	Weekly	12 taught sessions	20 mins	Postures
Gallagher et al. 2020	USA	RCT high-risk pregnancy on bedrest	79 (48/31)	23–31 weeks	Yoga sessions	Standard care and no yoga	Depression Anxiety	Perceived anxiety and depression overall scores lower in yoga group than in control group (*p* < 0.001)	Bi-weekly taught, video self-practice	Average of 7.46 (3–16) taught sessions, and 2 (0–24) self-practice video sessions	30 mins	Breathing, visulisation, adapted yoga moves, yoga nidra
Hayase et al. 2018	Japan	Non-randomised control trial uncomplicated pregnancy	91 (38/53)	20–23 weeks	Yoga sessions	Standard antenatal care	Perceived stress Physiological stress	PSS scores lower in yoga group at 20–23 & 28–31 weeks’ gestation. Salivary α-amylase levels in yoga group significantly decreased immediately after yoga	Weekly taught and daily self- practice	Average of 4 to 19 taught sessions and all women practiced yoga for > 15 min at home, at least three times a week based on the self-report	60 mins taught session and 15 mins self- practice	Warm-up, breathing exercises, postures, meditation
Jahdi et al. 2017	Iran	RCT normal pregnancy	60 (30/30)	26 weeks	Yoga sessions	Routine midwifery care	Mode of birth Duration of labour Pain management	Duration of the second and third stages of labour significantly shorter in yoga group (*p* = 0.04 and 0.01 respectively). Caesarean section rate 13.3% in yoga group compared to 50% in control group. Analgesic use during first stage of labour showed no difference between groups (*p* = 0.2)	Tri-weekly taught & daily self-practice	33 taught sessions and possibility of 44 self- practice sessions	60 mins	Postures, chanting om, breath awareness, yoga nidra, dhyana
Kundarti et al. 2020	Indonesia	RCT normal pregnancy	59 (30/29)	20–32 weeks	Yoga sessions	Standard antenatal care	Anxiety Physiological stress	Average anxiety in the intervention and control group after intervention (M = 13.16) vs (M = 35.30) and average cortisol levels (M = 16.50) vs (M = 9.91)	Weekly	8 taught sessions	90 mins	Postures, breathing, meditation shavasana
Makhija et al. 2021	India	RCT mild hypertensive disorder pregnancy	60 (30/30)	Third trimester	Integrated yoga	Routine care	Duration of labour Mode of birth	Reduction in total duration of labour in yoga group (*p* = 0.011). 22 (73.3%) yoga group had vaginal delivery compared to 18 (60%) in control group	Tri-weekly	At least 4 weeks (12 sessions)	40 mins	Postures, breathing, meditation
Mitchell et al. 2012	USA	RCT depression	24 (12/12)	20 weeks	Yoga postures	Parenting education sessions	Depression	Depressive symptoms reduced to subclinical levels in 55% of yoga group compared to 11% of control group	Bi-weekly	24 taught sessions	20 mins	Postures
Mohyadin et al. 2020	Iran	RCT normal pregnancy	84 (42/42)	26–37 weeks	Yoga sessions	Routine midwifery care	Anxiety Pain management Duration of labour Mode of birth	Anxiety lower in yoga group (*p* = 0.003). less pain at 4-5 cm in yoga group (*p* = 0.001). Shorter duration of labour in yoga group (*p* = 0.003)	Bi-weekly taught and tri-weekly home practice	6 sessions	60 mins	Postures, breathing, meditation
Munirekha et al. 2019	India	True-experimental post-test only control group design - uncomplicated pregnancy	30 (15/15)	24–32 weeks	Yoga sessions	Health education on antenatal care and future lactation	Mode of birth	NVB 80% yoga group compared to 40% control group	Weekly	Taught from 24 to 32 weeks until delivery	No information	Yogasanas
Narendran et al. 2005	India	Non-randomised control trial normal pregnancy	335 (169/166)	18–20 weeks	Yoga therapy	Walking 30 mins twice daily	Mode of birth	NVB 54% yoga group compared to 49% control group	Daily	Mean GA at delivery 38 weeks allowing for availability of 126 sessions	60 mins	Integrated approach of yoga therapy (IAYT) Taught then self-practice
Newham et al. 2014	UK	RCT healthy pregnant women	59 (31/28)	20–24 weeks	Yoga sessions	TAU	Anxiety Depression Physiological stress	Greater reduction in both anxiety and depression in the yoga group. Significant decrease in cortisol after yoga (0.15 [0.11]μg/dL vs. 0.13[0.10]μg/dL *P* = 0.003)	Weekly	8 taught sessions	1.5 h	Hatha yoga
Rakhshani et al. 2010	India	RCT normal pregnancy	102 (51/51)	18–20 weeks	Integrated yoga	Standard antenatal exercises	Quality of life	Between groups analysis showed significant improvements in the yoga group in the physical (*P* = 0.001), psychological (*P* = 0.001), social (*P* = 0.003) environmental domains (*P* = 0.001) of the WHOQOL-100	Tri-weekly	If until delivery estimated between 54 and 66 available taught sessions	60 mins	Lectures, breathing exercises, postures, meditation, deep relaxation
Rakhshani et al. 2012	India	RCT high-risk pregnancy	68 (30/38)	12 weeks	Integrated yoga	Standard care plus walking for half an hour mornings and evenings	Mode of birth	Lower rate of emergency c-section in yoga group 51.7% compared to 57.9% in control	Tri-weekly	28 taught sessions	60 mins	Breathing exercises, yogic postures, meditative exercises
Rong et al. 2021	China	RCT normal healthy pregnancy	64 (32/32)	18–27 weeks	Yoga sessions	Routine prenatal care	Anxiety Depression Duration of labour Mode of birth	No statistically significant difference in post anxiety and depressions scores. Higher rate of vaginal birth (*p* = 0.039) and shorter duration of labour (*p* = 0.002) in yoga group	Tri-weekly	12 weeks (up to 36 sessions)	60 mins	Warm-up, postures, meditation
Ruqaiyah et al. 2020	India	Quasi-experimental pre/post with control	24 (12/12)	Third trimester	No information	No information	Anxiety	Lower anxiety in the yoga group post intervention (*p* = 0.002)	No information	No information	No information	No information
Satyapriya et al. 2009	India	RCT normal pregnancy	90 (45/45)	18–20 weeks	Integrated yoga	Standard prenatal exercise	Perceived stress	Perceived stress decreased by 31.57% in the yoga group and increased by 6.60% in the control group (*P* = 0.001)	Tri-weekly for first month then daily self- practice	1 month taught then self- practice, refresher every 4 weeks until 28 weeks then 2 weeks until 36 weeks. 16-week programme with availability of up to 92 sessions	120 mins taught, 60 mins self-practice	Lectures, breathing exercises, poses, meditation, deep relaxation
Satyapriya et al. 2013	India	RCT normal pregnancy	96 (51/45)	18–20 weeks	Integrated yoga	Standard antenatal exercises	Anxiety Depression Perceived stress	Anxiety and Depression reduced with improvement in pregnancy experience in the yoga group (*P* < 0.001)	Tri-weekly for first month then daily self- practice	16-week programme estimated up to 92 available sessions	120 mins taught, 60 mins self-practice	Lectures, breathing exercises, poses, deep relaxation, meditation
Uebelacker et al. 2016	USA	RCT depression	20 (12/8)	12–26 weeks	Yoga sessions	Mom-baby wellness workshops	Depression	Although both groups had reduced depression scores, yoga was preferred.	Weekly	9 taught sessions & self- practice	75 mins	Breathwork, warm-up, poses, relaxation, homework
Yekefallah et al. 2021	Iran	RCT normal pregnancy	70 (35/35)	26–37 weeks	Yoga sessions	Routine prenatal care	Duration of labour Mode of birth	Mean duration of labour was shorter in yoga group (*p* < 0.0001). 82.9% of the women in the yoga group and 65.7% in the control group had a natural delivery	Bi-weekly	Attended for 9–11 weeks (up to 22 sessions)	75 mins	Hatha yoga
Yulianti et al. 2018	Indonesia	RCT normal pregnancy	102 (51/51)	22–32 weeks	Yoga sessions	Not treated	Depression Anxiety	Mean level of anxiety and depression were lower in the yoga group at both two- and four-weeks post intervention (*p* < 0.001)	No information	1 month	No information	No information
Yuvarani et al. 2020	India	Quasi-experimental pre/pots with control depression and anxiety	30 (15/15)	16–20 weeks	Yoga sessions	Aerobic exercise	Depression	Aerobic exercise and yoga showed significant effect for reducing the symptoms of depression (*P* ≤ 0.001)	Weekly	3 months (up to 13 sessions)	20 mins	Breathing, postures

### Characteristics of pregnancy yoga interventions

The frequency of the pregnancy yoga intervention ranged from a single session to daily, session length ranged from 20to 120 min and intensity ranged from a single session to availability of 126 practice sessions. Four studies classified the yoga intervention as yoga therapy [28, 30, 34, 35], eighteen yoga sessions [8, 11, 33, 36, 39, 43–55], three yoga postures [40–42], five integrated yoga therapy [13, 29, 31, 32, 37] and one did not provide details [38]. All yoga interventions used physical postures. Of the 31 included studies, 27 did not define the specific style of yoga used in the intervention; three cited hatha yoga [43, 48, 55] and one Ashtanga Vinyasa [44].

### Risk of bias

**Fig. 2:**
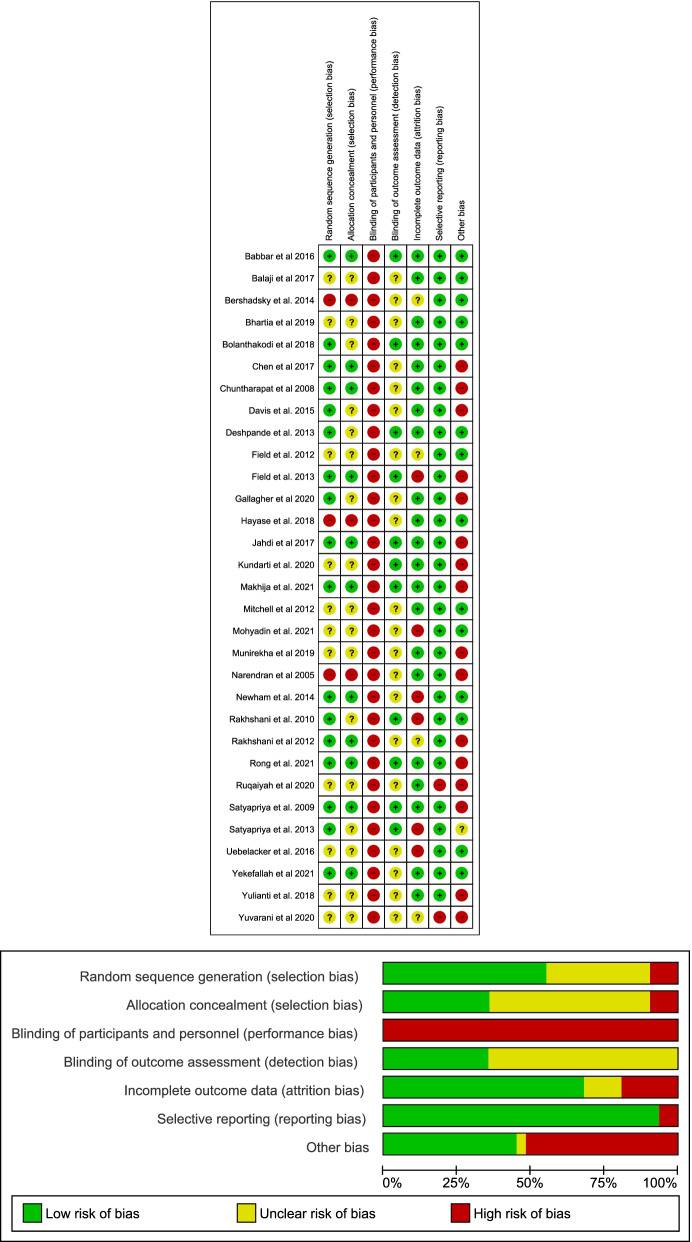
Summary of Risk of Bias and Risk of bias for individual studies

All studies were assessed as having a high-risk of bias for at least one domain. The overall risk of bias assessment across domains and the risk of bias in each included study are displayed in Fig. 2. Sixteen studies were rated high-risk of other bias due to exclusion of participants from the final analysis without explanation, baseline imbalances, loss to follow-up imbalances, self-selection bias, self-reports of compliance, lack of clarity on the administration of the yoga intervention and use of insensitive instruments to measure outcomes.

### Assessment of the quality of the evidence - GRADE

The quality assessment for individual review outcomes informed by the GRADEpro Guideline Development Tool (GDT) are reported in Table 2. There was low quality evidence that pregnancy yoga interventions could be effective for each outcome included in this review.

**Table 2:** Summary of findings

**Yoga for pregnancy**						
**Patient or population:** pregnant women **Settings:** Any **Intervention:** yoga **Comparison:** treatment as usual or any other active treatment						
**Outcomes**	Illustrative comparative risks* (95% CI)		**Relative effect** **(95% CI)**	**No of Participants** **(studies)**	**Quality of the evidence** **(GRADE)**	**Comments**
	Assumed risk	Corresponding risk				
	**Treatment as usual or any other active treatment**	**Yoga**				
**Anxiety** STAI, HADS-A, Hamilton Follow-up: 2-18 weeks		The mean anxiety in the intervention groups was **0.91 standard deviations lower** (1.49 to 0.33 lower)		733 (11 studies)	⊕⊕⊝⊝ **low** ^1,2,3,4,5^	SMD -0.84 (-1.64 to -0.03)
**Depression** CES-D, HADS-D, Hamilton, EPDS Follow-up: 2-18 weeks		The mean depression in the intervention groups was **0.47 standard deviations lower** (0.90 to 0.04 lower)		679 (12 studies)	⊕⊕⊝⊝ **low** ^1,2,3,4,5,6,7^	SMD -0.53 (-1.04 to -0.02)
**Perceived stress** PSS-10; Pregnancy experiences questionnaire (PEQ) Follow-up: 12-24 weeks		The mean perceived stress in the intervention groups was **1.03 standard deviations lower** (1.55 to 0.52 lower)		423 (5 studies)	⊕⊕⊝⊝ **low** ^1,2,5^	
**Physiological stress** Salivary cortisol Follow-up: 4-20 weeks		The mean physiological stress in the intervention groups was **0.69 standard deviations lower** (1.50 lower to 0.13 higher)		279 (4 studies)	⊕⊝⊝⊝ **very low** ^1,2,3,4,5,6,8,9^	
**Total duration of labour** medical records Follow-up: 10-24 weeks		The mean total duration of labour in the intervention groups was **117.75 lower** (153.80 to 81.71 lower)		472 (6 studies)	⊕⊕⊝⊝ **low** ^1,2,3,8^	
**Normal vaginal birth** medical records Follow-up: 10-28 weeks	**Study population**		**OR 2.58** (1.46 to 4.56)	1195 (12 studies)	⊕⊝⊝⊝ **very low** ^1,2,3,5,6,10^	
	**51 per 100**	**73 per 100** (61 to 83)				
	**Moderate**					
	**49 per 100**	**72 per 100** (59 to 82)				
**Quality of life** WHOQoL100 Follow-up: mean 16 weeks		The mean quality of life in the intervention groups was **1.73 higher** (0.79 to 2.67 higher)		102 (1 study)	⊕⊕⊝⊝ **low** ^1,2,8^	

*The basis for the **assumed risk** (e.g., the median control group risk across studies) is provided in footnotes. The **corresponding risk** (and its 95% confidence interval) is based on the assumed risk in the comparison group and the **relative effect** of the intervention (and its 95% CI)

**CI:** Confidence interval; **OR:** Odds ratio

GRADE Working Group grades of evidence

**High quality:** Further research is very unlikely to change our confidence in the estimate of effect

**Moderate quality:** Further research is likely to have an important impact on our confidence in the estimate of effect and may change the estimate

**Low quality:** Further research is very likely to have an important impact on our confidence in the estimate of effect and is likely to change the estimate

**Very low quality:** We are very uncertain about the estimate.

^1^Concerns with high-risk of bias for allocation concealment

^2^Concerns with high-risk of bias for lack of blinding of participants

^3^Concerns with high-risk of bias due to unclear evidence on blinding of outcome assessors

^4^Serious inconsistency due to large variation in effect across studies

^5^Serious inconsistency I2 value is large indicating substantial heterogeneity

^6^Concerns with high-risk of bias for random allocation

^7^Concerns with high-risk of bias due to pre-existing depression or depressive symptoms in some studies

^8^Serious imprecision based on total population size >400

^9^Serious imprecision due to wide 95% CI's

^10^Serious inconsistency due to inclusion of high risk pregnant populations

*Abbreviations*: *STAI* state and trait anxiety scale, *HADS-A* hospital anxiety and depression scale - anxiety, *HAM-A* Hamilton anxiety rating scale, *CES-D* centre for epidemiological studies - depression, *HADS-D* hospital anxiety and depression scale - depression, *HDRS* Hamilton depression rating scale, *EPDS* Edinburgh postnatal depression scale, *PSS-10* perceived stress scale 10 item, *PEQ* pregnancy experiences questionnaire

### Primary outcomes

#### Stress

**Fig. 3:**
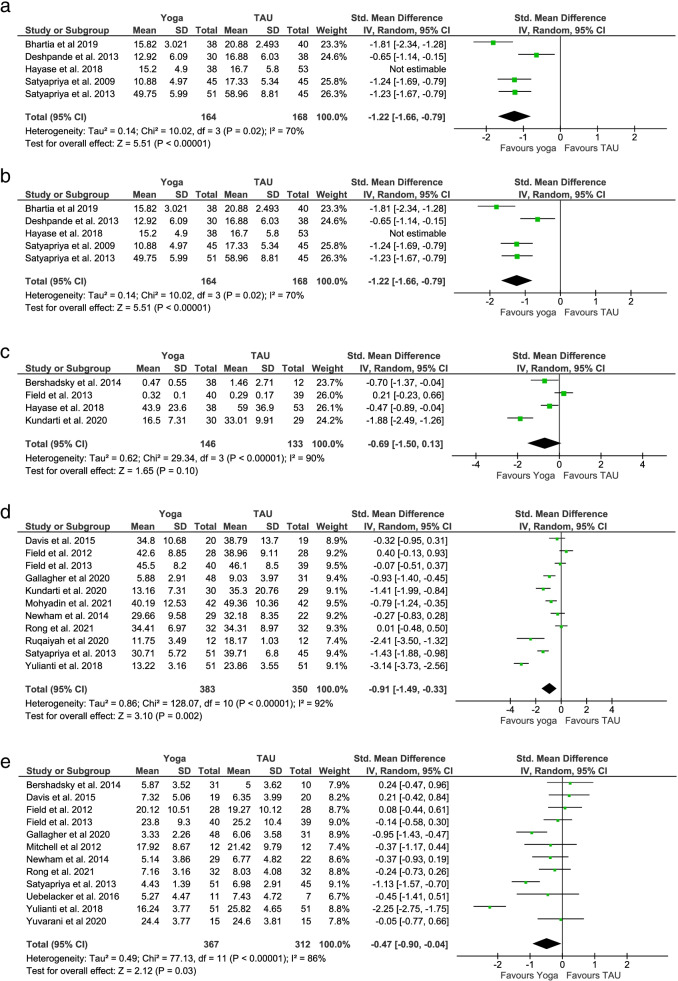
Meta-analysis primary outcomes

Five RCTs with 423 participants reported post-intervention perceived stress scores measured by the Perceived Stress Scale (PSS-10) [13, 28, 35, 53] and the Pregnancy Experiences Questionnaire (PEQ) [29]. The pooled SMD (− 1.03; 95% CI: − 1.55 to − 0.52; *p* < 0.001) supports a statistically significant beneficial effect of pregnancy yoga interventions for perceived stress (Fig. 3a). A sensitivity analysis removing a study at high risk of bias supported these results and lowered heterogeneity (Tau^2^ = 0.14, I^2^ = 70%; p < 0.001) [53] (Fig. 3b). Four RCTs with 279 participants reported post-intervention stress levels, measured by salivary or plasma cortisol [41, 43, 52, 53]. The pooled SMD (− 0.69; 95% CI: − 1.50 to 0.13; *p* = 0.10) demonstrated no significant effect for physiological stress (Fig. 3c). A further two RCTs reported data on physiological stress but were not suitable for meta-analysis [49, 55]. Chen et al. looked at short-term and long-term stress and immunological effects of yoga in 94 healthy pregnant women [49]. Although yoga displayed a short-term decrease in cortisol, there were no significant differences in long-term cortisol effects between groups. The second RCT conducted by Newham et al. with 29 pregnant women reported that salivary cortisol levels were significantly lower immediately after the yoga intervention [55].

#### Anxiety

Eleven RCTs with 733 participants reported post-intervention anxiety symptom scores measured by the State-Trait Anxiety Inventory (STAI), Hospital Anxiety and Depression Scale – Anxiety (HADS-A) and Hamilton Anxiety Rating Scale (HAM-A) [29, 38, 40, 41, 44, 45, 47, 50–52, 55]. The pooled SMD (− 0.91; 95% CI: − 1.49 to − 0.33; *p* = 0.002) supports a statistically significant beneficial effect of pregnancy yoga interventions for anxiety (Fig. 3d).

#### Depression

Twelve RCTs with 679 participants reported post-intervention depression symptom scores measured by Centre for Epidemiological Studies - Depression (CES-D), Hospital Anxiety and Depression Scale - Depression (HADS-D), Edinburgh Postnatal Depression Scale (EPDS) and Hamilton Depression Rating Scale (HDRS) [11, 29, 39–45, 50, 51, 55]. The pooled SMD (− 0.47; 95% CI: − 0.90 to − 0.04; *p* = 0.03) supports a statistically significant beneficial effect of pregnancy yoga interventions for depression symptoms (Fig. 3e). Sensitivity analysis performed afterremoval of one study with high risk of bias from the analysis showed no difference [51].

#### Quality of life

One RCT with 102 participants reported post-intervention quality of life scores measured by the World Health Organization Quality of Life Assessment Instrument (WHOQoL-100) [31]. Between-group analysis showed significant improvements in the yoga group compared to the control in the physical (15.79 ± 2.77 (15–16.570, *p* = 0.001), psychological (16.08 ± 2.12 (15–16.57), *p* < 0.001), social relationships (16.88 ± 1.91 (16.34–17.42), *p* = 0.003) and environmental domains (16.25 ± 2 (15.69–16.82), p = 0.001). Results were not significant for independence (15.91 ± 2.2 (15.29–16.53), *p* = 0.065) and spiritual domains (16.02 ± 2.42 (15.34–16.70), *p* = 0.23).

### Secondary outcomes

#### Labour duration

**Fig. 4:**
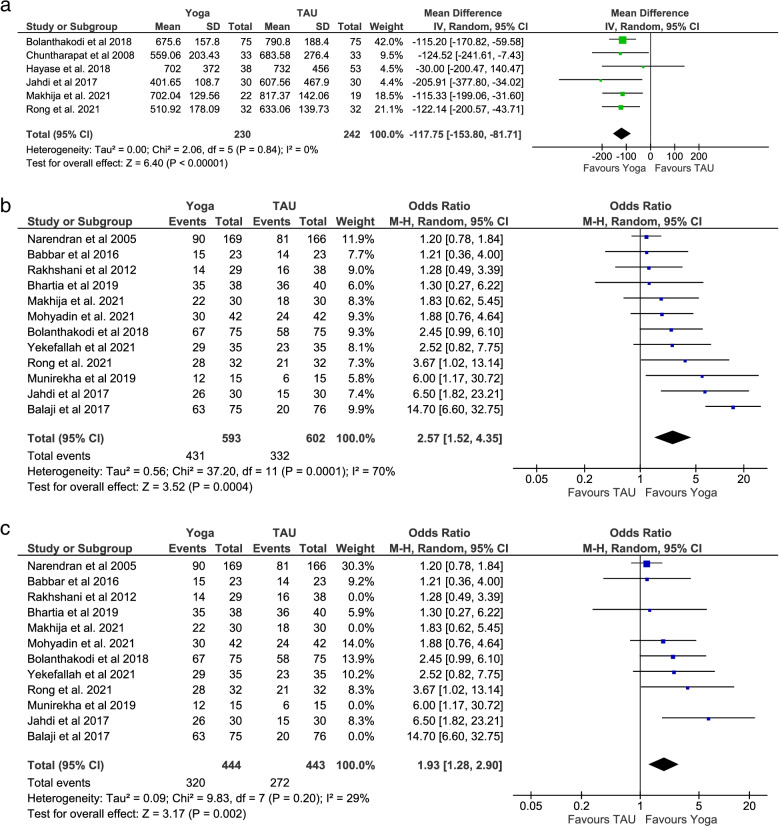
Meta-analysis secondary outcomes

Six RCTs with 472 participants reported data on the duration of labour [34, 37, 46, 50, 53, 54]. The pooled MD calculated in minutes (− 117.75; 95% CI: − 153.80 to − 81.71; *p* < 0.001) supports a statistically significant beneficial effect of pregnancy yoga interventions for shorter duration of labour by an average of almost 2 h (Fig. 4a). Sensitivity analysis performed after removal of one study with high risk of bias from the analysis showed no difference [53].

#### Pain management

Four RCTs with 360 participants reported data on pain management during labour [34, 46, 47, 54]. Data from these studies were not suitable for meta-analysis. One study demonstrated a significant reduction in requirements for intravenous analgesia in the pregnancy yoga group (*p* < 0.045). Tolerance of pain measured by the Numerical Pain Intensity Scale (NPIS) (*p* < 0.001) and Pain Behavioural Observation Scale (PBOS) was also increased in the pregnancy yoga group (*p* < 0.001) [34]. A second study found that the pregnancy yoga group demonstrated significantly higher maternal comfort during labour, measured by the Visual Analogue Sensation of Pain Scale (VASPS) and PBOS (*p* < 0.05), while no differences were found between the groups forpethidine usage [54]. A third study found that analgesic use during the first stage of labour showed no difference between groups (*p* = 0.2) [46] and the fourth study reported that the mean pain score at 4-5 cm cervical dilatation was significantly lower in yoga intervention group (*p* = 0.001) [47].

#### Mode of birth

Twelve studies with 1195 participants reported data on the mode of birth [8, 30, 32–37, 46–48, 50]. Compared to control groups the vaginal birth rate was significantly higher in the pregnancy yoga groups (OR = 2.57; 95% Cl: 1.52–4.35; *p* < 0.001) (Fig. 4b). Sensitivity analysis performed after removal of four studies with a focus on high-risk pregnancies, with an implied increased risk of a caesarean birth, from the analysis maintained an increased likelihood of a vaginal birth in the pregnancy yoga group (OR = 1.93; 95% Cl: 1.28–2.90; *p* = 0.002) [32, 33, 36, 37] (Fig. 4c). As expected, removing these studies also reduced heterogeneity (Tau^2^ = 0.09, I^2^ = 29%; p = 0.002 compared to Tau^2^ = 0.56, I^2^ = 70%; *p* < 0.001).

### Subgroup FITT principle of exercise prescription analysis

**Fig. 5:**
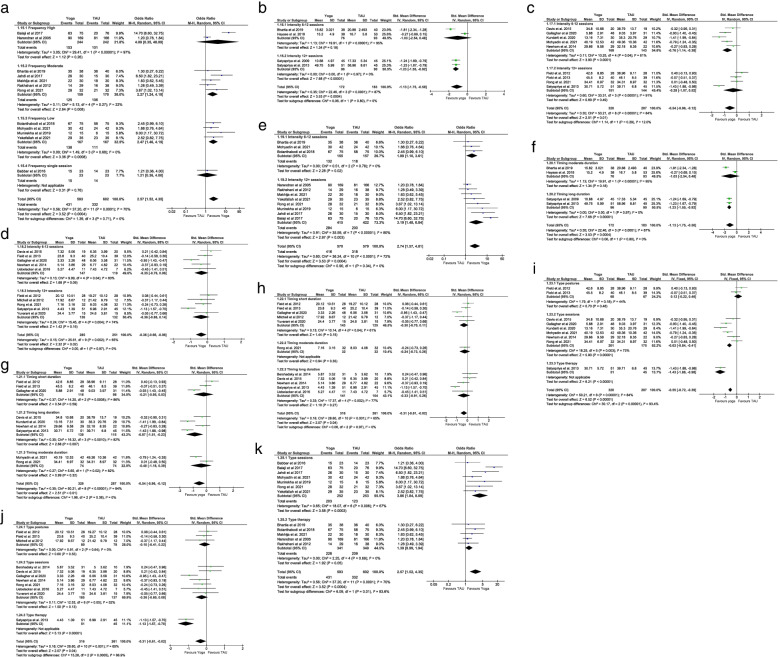
Subgroup FITT principle analysis of study outcomes

The FITT principle of exercise prescription was applied across studies and detailed results are reported in Additional file 4 and Fig. 5.

#### Frequency

The test for subgroup differences for mode of birth suggest that there is a statistically significant subgroup effect for low-frequency yoga interventions of weekly or bi-weekly sessions on mode of birth (*p* < 0.001) (Fig. 5a).

#### Intensity

There was a statistically significant subgroup effect on perceived stress for interventions with more than 12 sessions (p < 0.001) (Fig. 5b), while 6–12 sessions had the most significant impact on anxiety (*p* < 0.001) (Fig. 5c). For depression, there was no statistically significant difference for interventions with 6–12 sessions (*p* = 0.09) or more than 12 sessions (*p* = 0.16) (Fig. 5d). Interventions with more than 12 sessions had a statistically significant positive impact on the rate of normal vaginal births (*p* = 0.003) (Fig. 5e).

#### Time

Long-duration yoga interventions greater than 60 min had a statistically significant positive effect on perceived stress (*p* < 0.001) and anxiety (*p* = 0.007) (Fig. 5f & g). There was no statistically significant difference on depression scores between short (*p* = 0.15), moderate (*p* = 0.35) and long duration yoga interventions (*p* = 0.27) (Fig. 5 h).

#### Type

There was a statistically significant subgroup effect for yoga sessions (*p* < 0.001) and yoga therapy (*p* < 0.001) compared to yoga postures (*p* = 0.48) on anxiety (Fig. 5i). The analysis for depression indicates a statistically significant subgroup effect for yoga therapy (*p* < 0.001) (Fig. 5j) while there was a statistically significant subgroup effect for yoga sessions on mode of birth (*p* < 0.001) (Fig. 5 k).

## Discussion

This systematic review examined the published evidence on pregnancy yoga to explore the characteristics and effectiveness of pregnancy yoga interventions. Notably only four studies specifically named a type of yoga. The frequency, intensity, duration and content of the interventions varied widely. Encouragingly, results of the meta-analysis suggest that yoga is a beneficial non-pharmacological intervention to manage levels of stress, anxiety and depression in pregnant women. In relation to birth outcomes, meta-analysis showed that women in the yoga groups experienced shorter duration of labour up to 2 h on average, were 2.5 times more likely to experience a normal vaginal birth, had reduced intravenous analgesic administered and reported higher levels of comfort. Optimistically, low-frequency yoga interventions had a more significant impact on mode of birth while interventions with 6–12 sessions reduced anxiety.

These findings are supported by a previous qualitative review that examined yoga and its efficacy with 10 of the 15 studies demonstrating positive changes in maternal psychological or birth outcome measures [56]. A recent meta-analysis also found that yoga was an effective complementary and alternative therapy in promoting vaginal births and shortening the first and second stages of labour [16]. Notably, other studies have reported clinically meaningful changes in pain management for a multitude of conditions following yoga [57–60]. There is however a paucity of research in the area and further understanding of the mechanisms by which yoga can influence and modify the pain response is needed. Of the 31 included studies, 13 were conducted in India and a recent systematic review demonstrated that RCTs on yoga that were conducted in India were about 25 times more likely to reach positive conclusions than those conducted elsewhere [57]. Further in-depth studies are recommended to elucidate reasons for differences in conclusions between yoga RCTs conducted in India and those conducted elsewhere, and it may be beneficial to report on the results of trials conducted in India separately in future reviews. Since India is considered the home of yoga perhaps there are inherent differences in how yoga is taught and practised and how it is perceived by its population.

Of note we found no evidence of adverse events in any of the trials, suggesting that yoga is a safe practice during pregnancy. According to Mottola & Artal (2016), in order to provide safe exercise guidelines, pregnant women should be prescribed exercises in accordance with the FITT principle [61]. Future studies should focus on specifying the frequency, intensity, duration and type of yoga in order to better understand the components of the intervention that impact optimally on both pregnancy outcomes and safety. This could then facilitate the development of a checklist of essential components for an evidence-based pregnancy yoga practice that could be replicated. The review results highlight issues regarding lack of allocation concealment and double-blinding, attrition bias, small sample sizes, a wide variety of outcome measures, non-standardised or replicable yoga interventions, lack of measurement of fidelity to the intervention and huge variation in the components of the yoga interventions. Many studies used self-practice which is difficult to monitor for both compliance and safety. High levels of compliance and safety are important for interventions to be effective so future studies should consider how the intervention is delivered and monitored. This will improve fidelity and potentially maximise effect. This is the first meta-analysis to suggest the optimal number and frequency of sessions to maximise effect and future trials can use these data to plan sessions numbers and frequency of delivery based on their intended outcomes. Importantly, women in the included studies were of middle-to-high socioeconomic status, presenting a selection bias of participants and thus reducing generalisability. Further studies should be conducted with women from lower socio-economic backgrounds.

A strength of this study is that the protocol was registered on PROSPERO and published open access. It followed the PRISMA statement, evaluated the certainty of the evidence using the GRADE methodology and all results were continuously reviewed by at least two reviewers. The findings can support the incorporation of the FITT principle into the design of interventions for future pregnancy yoga trials. In terms of limitations, inclusion of only quantitative studies published in English might have excluded those published in other languages and/or qualitative studies. While the Peer Review of Electronic Search Strategies (PRESS) for systematic reviews was not used a wide variety of databases were searched and a subject librarian supported the process of structuring and optimising the search strategy.

## Conclusion

The present review and meta-analysis offer valuable information on the characteristics and effectiveness of pregnancy yoga interventions. The evidence supports previously cited positive effects of pregnancy yoga on anxiety, depression, perceived stress, normal vaginal birth and shorter duration of labour. Recommendations above can be used to support researchers to work collaboratively with yoga practitioners to standardise pregnancy yoga interventions and conduct more robust evidence-based evaluation. Overall, the evidence supporting yoga in pregnancy is growing, but methodological weaknesses with published studies and an insufficient number of published RCTs with reproducible evidence-based interventions highlight the need for further research. More high-quality studies are needed before the efficacy of pregnancy yoga interventions for maternal and birth outcomes can be definitively known. Future studies should ensure rigorous trial design and reporting alongside evidence-informed intervention development.

## Supplementary Information

**Additional file 1.** PRISMA Checklist.

**Additional file 2.** PICOS and Search strategy.

**Additional file 3.** Data extraction form.

**Additional file 4.** Subgroup FITT principle of exercise prescription analysis.

## Data Availability

The dataset generated and/or analysed during the study are available from the corresponding author on reasonable request.

## Abbreviations

ADHD: Attention deficit hyperactivity disorder
CES-D: Centre for epidemiological studies - depression
CI: Confidence interval
EPDS: Edinburgh postnatal depression scale
FITT: Frequency, intensity, time/duration and type
GRADE: Grades of recommendation, assessment, development and evaluation
HADS-A: Hospital anxiety and depression scale - anxiety
HADS-D: Hospital anxiety and depression scale - depression
HAM-A: Hamilton anxiety rating scale
HDRS: Hamilton depression rating scale
OR: Odds ratio
PIH: Pregnancy induced hypertension
PRISMA-P: Preferred reporting items for systematic reviews and meta-analysis protocols
PSS-10: Perceived stress scale 10 item
QoL: Quality of life
RCT: Randomised control trial
RR: Risk ratio
STAI: The state-trait anxiety inventory
WHOQoL-100: World health organisation quality of life assessment instrument
