# Supplementary material for: The characteristics and effectiveness of pregnancy yoga interventions: a systematic review and meta-analysis
Source: BMC Pregnancy Childbirth. 2022 Mar 25;22:250. doi: 10.1186/s12884-022-04474-9 (PMC8957136; doi:10.1186/s12884-022-04474-9)
Supplement: Supplementary file 2 — Additional file 2. PICOS and Search strategy. [file 12884_2022_4474_MOESM2_ESM.docx]

**Additional file 2:**

**Search terms used in PICOS search**

| PICOS | Definition | Search keywords |
| --- | --- | --- |
| Population | Pregnant women | pregnan* OR gestat* OR “expectant mother*” OR “pregnant woman” OR “pregnant women” OR prenatal OR antenatal OR pre-natal OR ante-natal OR “first time mother*” OR “first-time mother*” OR “first-time mum*” OR post-natal OR postnatal OR postpartum OR post-partum |
| Intervention | Yoga for pregnancy | yoga* |
| Comparator | Treatment as usual or any active treatment other than yoga | Not set |
| Outcomes | Stress, anxiety, depression, quality of life, labour duration, pain management in labour, mode of birth | Not set |
| Study design | RCTs or quasi-experimental studies | Not set |

**Search strategy for EMBASE 29 July 2019**

Concept 1: Pregnancy

 EMBASE: 'pregnancy'/exp OR 'expectant mother'/exp

Keywords (title & abstract): pregnan* OR gestat* OR “expectant mother*” OR “pregnant woman” OR “pregnant women” OR prenatal OR antenatal OR pre-natal OR ante-natal OR “first time mother*” OR “first-time mother*” OR “first-time mum*” OR post-natal OR postnatal OR postpartum OR post-partum

Concept 2: Yoga

 EMBASE: 'yoga'/exp

Keywords (title & abstract): **yoga***


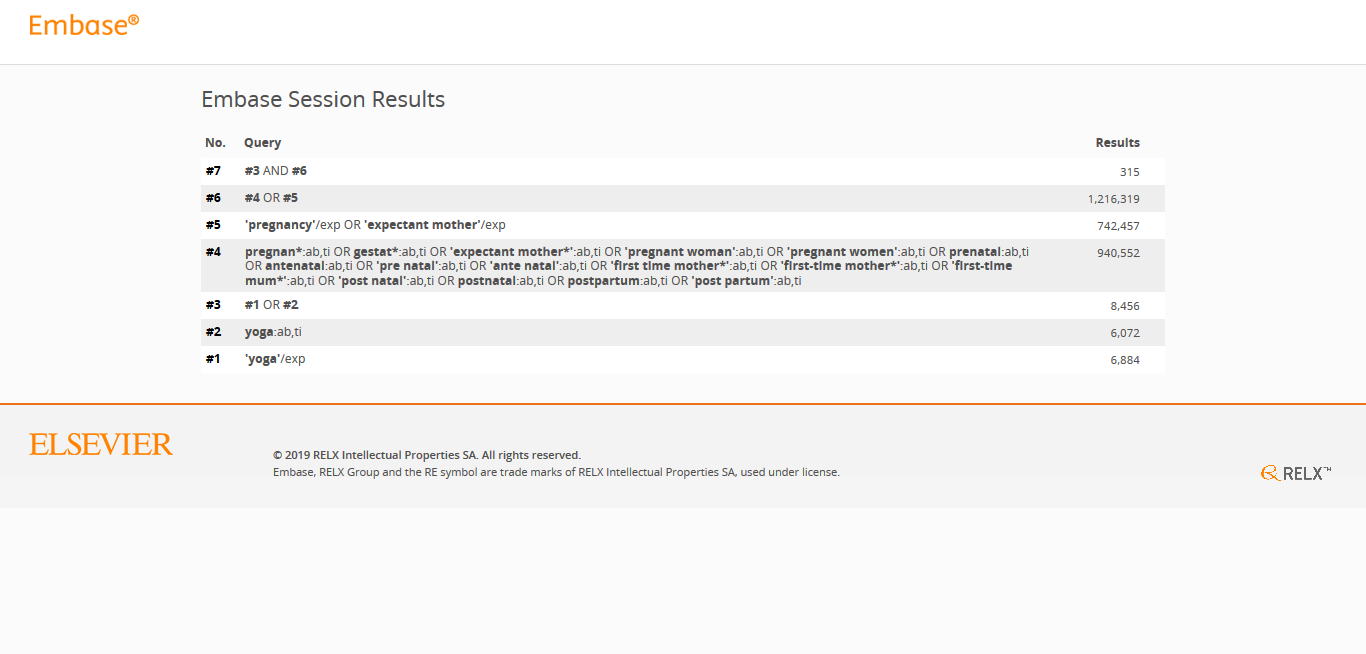


**Search Queries**

| **No.** | **Query** | **Results** | **Date** |
| --- | --- | --- | --- |
| #7 | ('yoga'/exp OR yoga:ab,ti) AND ((pregnan*:ab,ti OR gestat*:ab,ti OR 'expectant mother*':ab,ti OR 'pregnant woman':ab,ti OR 'pregnant women':ab,ti OR prenatal:ab,ti OR antenatal:ab,ti OR 'pre natal':ab,ti OR 'ante natal':ab,ti OR 'first time mother*':ab,ti OR 'first-time mother*':ab,ti OR 'first-time mum*':ab,ti OR 'post natal':ab,ti OR postnatal:ab,ti OR postpartum:ab,ti OR 'post partum':ab,ti) OR ('pregnancy'/exp OR 'expectant mother'/exp)) | 315 | 29 Jul 2019 |
| #6 | (pregnan*:ab,ti OR gestat*:ab,ti OR 'expectant mother*':ab,ti OR 'pregnant woman':ab,ti OR 'pregnant women':ab,ti OR prenatal:ab,ti OR antenatal:ab,ti OR 'pre natal':ab,ti OR 'ante natal':ab,ti OR 'first time mother*':ab,ti OR 'first-time mother*':ab,ti OR 'first-time mum*':ab,ti OR 'post natal':ab,ti OR postnatal:ab,ti OR postpartum:ab,ti OR 'post partum':ab,ti) OR ('pregnancy'/exp OR 'expectant mother'/exp) | 1216319 | 29 Jul 2019 |
| #5 | 'pregnancy'/exp OR 'expectant mother'/exp | 742457 | 29 Jul 2019 |
| #4 | pregnan*:ab,ti OR gestat*:ab,ti OR 'expectant mother*':ab,ti OR 'pregnant woman':ab,ti OR 'pregnant women':ab,ti OR prenatal:ab,ti OR antenatal:ab,ti OR 'pre natal':ab,ti OR 'ante natal':ab,ti OR 'first time mother*':ab,ti OR 'first-time mother*':ab,ti OR 'first-time mum*':ab,ti OR 'post natal':ab,ti OR postnatal:ab,ti OR postpartum:ab,ti OR 'post partum':ab,ti | 940552 | 29 Jul 2019 |
| #3 | 'yoga'/exp OR yoga:ab,ti | 8456 | 29 Jul 2019 |
| #2 | yoga:ab,ti | 6072 | 29 Jul 2019 |
| #1 | 'yoga'/exp | 6884 | 29 Jul 2019 |
