# Supplementary material for: The characteristics and effectiveness of pregnancy yoga interventions: a systematic review and meta-analysis
Source: BMC Pregnancy Childbirth. 2022 Mar 25;22:250. doi: 10.1186/s12884-022-04474-9 (PMC8957136; doi:10.1186/s12884-022-04474-9)
Supplement: Supplementary file 3 — Additional file 3. Data extraction form. [file 12884_2022_4474_MOESM3_ESM.docx]

**Additional file 3:**


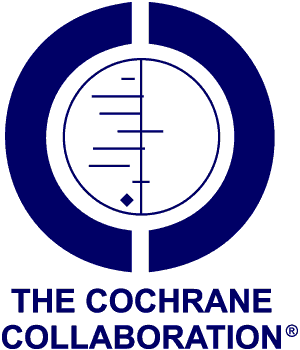


Data Extraction form

**Title of Systematic Review:**  Systematic review of the components and effectiveness of pregnancy yoga

**Trial Registration:** https://www.crd.york.ac.uk/prospero/display_record.php?RecordID=119916

This form has been developed by adopting and customising the “Data collection form for intervention review – RCT and non-RCTs” of the Cochrane Collaboration. Sections have been expanded and added, and irrelevant sections removed. Information included on this form should be comprehensive and may be used in the text of the review.

Notes on using a data extraction form:

- Be consistent in the order and style you use to describe the information for each included study.
- Record any missing information as unclear or not described, to make it clear that the information was not found in the study report(s), not that you forgot to extract it.
- Include any instructions and decision rules on the data extraction form, or in an accompanying document. It is important to practice using the form and give training to any other authors using the form.

**General Information**

| Date form completed |  |
| --- | --- |
| Name/ID of person extracting data |  |
| Article Title |  |
| Study author contact details |  |
| Publication type |  |
| Country in which the study was conducted |  |
| Study funding source (including role of funders) |  |
| Possible conflicts of interest |  |
| Notes: | |

**Study eligibility**

| Study Characteristics | Review Inclusion Criteria   - Written in English - Original article - Intervention study describing a yoga class intervention - Intervention includes yoga poses and practices - Randomised and quasi-experimental studies - Studies that compared yoga with usual care or any active treatment | | Inclusion criteria met? | | | Location in text or source *(pg & /fig/table/other)* |
| --- | --- | --- | --- | --- | --- | --- |
|  |  |  | Yes | No | Unclear |  |
| Type of study | Randomised Controlled Trial | | □ | □ | □ |  |
|  | Quasi-experimental study – control before and after, non-randomised control trials. | | □ | □ | □ |  |
| Participants |  | | □ | □ | □ |  |
| Types of intervention |  | | □ | □ | □ |  |
| Types of comparison |  | | □ | □ | □ |  |
| Types of outcome measures |  | | □ | □ | □ |  |
| INCLUDE □ | | EXCLUDE □ | | | | |
| Reason for decision |  | | | | | |
| Notes: | | | | | | |

**DO NOT PROCEED IF STUDY EXCLUDED FROM REVIEW**

Characteristics of included studies

**Methods**

|  | **Descriptions as stated in report/paper** | | **Location in text or source** *(pg & ¶/fig/table/other)* |
| --- | --- | --- | --- |
| **Aim of study** *(e.g. efficacy, effectiveness, feasibility)* |  | |  |
| **Design***(e.g. RCT, non-RCT describe)* |  | |  |
| **Unit of allocation/sampling technique** *(random assignment, convenience, step wedge, cluster)* |  | |  |
| **Start date** |  | |  |
| **End date** |  | |  |
| **Duration of participation** *(from recruitment to last follow-up)* |  | |  |
| **Ethical approval needed/ obtained for study** | □ □ □  Yes No Unclear |  |  |
| **Notes:** | | | |

**Participants**

|  | Description  *Include comparative information for each intervention or comparison group if available* | | Location in text or source *(pg & /fig/table/other)* |
| --- | --- | --- | --- |
| Population description *(from which study participants are drawn)* |  | |  |
| Setting *(including location and social context)* |  | |  |
| Inclusion criteria |  | |  |
| Exclusion criteria |  | |  |
| Method of recruitment of participants *(e.g. phone, mail, clinic, hospital)* |  | |  |
| Informed consent obtained | □ □ □  Yes No Unclear |  |  |
| Total no. randomised *(or total pop. at start of study for NRCTs)* |  | |  |
| Clusters *(if applicable, no., type, no. people per cluster)* |  | |  |
| Baseline imbalances |  | |  |
| Withdrawals and exclusions *(if not provided below by outcome)* |  | |  |
| Age (range) |  | |  |
| Race/Ethnicity |  | |  |
| Stage of pregnancy (weeks) |  | |  |
| Complications/comorbidities |  | |  |
| Other relevant socio-demographics (e.g. public, private healthcare, marital status) |  | |  |
| Notes: | | | |

**Risk of Bias assessment**

*See Chapter 8 of the Cochrane Handbook. Additional domains may be added for non-randomised studies.*

| Domain | Risk of bias | | | Support for judgement  *(include direct quotes where available with explanatory comments)* | Location in text or source *(pg & /fig/table/other)* |
| --- | --- | --- | --- | --- | --- |
|  | Low | High | Unclear |  |  |
| Random sequence generation *(selection bias)* | □ | □ | □ |  |  |
| Allocation concealment *(selection bias)* | □ | □ | □ |  |  |
| Blinding of participants and personnel*(performance bias)* | □ | □ | □ | Outcome group: All/ |  |
| *(if separate judgement by outcome(s) required)* | □ | □ | □ | Outcome group: |  |
| Blinding of outcome assessment *(detection bias)* | □ | □ | □ | Outcome group: All/ |  |
| *(if separate judgement by outcome(s) required)* | □ | □ | □ | Outcome group: |  |
| Incomplete outcome data *(attrition bias)* | □ | □ | □ | Outcome group: All/ |  |
| *(if separate judgement by outcome(s) required)* | □ | □ | □ | Outcome group: |  |
| Selective outcome reporting? *(reporting bias)* | □ | □ | □ |  |  |
| Other bias | □ | □ | □ |  |  |
| Notes: | | | | | |

**Intervention & Comparison groups**

*Copy and paste table for each intervention and comparison group*

**Intervention Group 1**

|  | Description as stated in report/paper | Location in text or source *(pg & ¶/fig/table/other)* |
| --- | --- | --- |
| Group name |  |  |
| No. randomised to group *(specify whether no. people or clusters)* |  |  |
| Description *(include sufficient detail for replication, e.g. content, dose, components)* |  |  |
| Duration of treatment period |  |  |
| Frequency *(e.g. frequency of each intervention, once a week, twice a week, daily)* |  |  |
| Intensity *(e.g. duration of each intervention or dose, 12 sessions, 24 sessions, 36 sessions)* |  |  |
| Timing *(e.g. length of each session, one hour. Two hours)* |  |  |
| Type *(e.g. Hatha, Vinyasa, Yoga Nidra, Pranayama, breathwork, relaxation, poses/asanas)* |  |  |
| Delivery *(e.g. mechanism, fidelity, location)* |  |  |
| Providers *(e.g. number, profession, training provided)* |  |  |
| Economic information *(i.e. intervention cost, changes in other costs as result of intervention)* |  |  |
| Resource requirements *(e.g. staff numbers, equipment, space)* |  |  |
| Integrity of delivery |  |  |
| Adherence/Attendance |  |  |
| Notes: | | |

**Comparison Group 1**

|  | Description as stated in report/paper | Location in text or source *(pg & ¶/fig/table/other)* |
| --- | --- | --- |
| Group name |  |  |
| No. randomised to group *(specify whether no. people or clusters)* |  |  |
| Description *(include sufficient detail for replication, e.g. content, dose, components)* |  |  |
| Duration of treatment period |  |  |
| Frequency *(e.g. frequency of each intervention, once a week, twice a week, daily)* |  |  |
| Intensity *(e.g. duration of each intervention or dose, 12 sessions, 24 sessions, 36 sessions)* |  |  |
| Timing *(e.g. length of each session, one hour. Two hours)* |  |  |
| Type *(e.g. Hatha, Vinyasa, Yoga Nidra, Pranayama, breathwork, relaxation, poses/asanas)* |  |  |
| Delivery *(e.g. mechanism, fidelity, location)* |  |  |
| Providers *(e.g. number, profession, training provided)* |  |  |
| Economic information *(i.e. intervention cost, changes in other costs as result of intervention)* |  |  |
| Resource requirements *(e.g. staff numbers, equipment, space)* |  |  |
| Integrity of delivery |  |  |
| Adherence/Attendance |  |  |
| Notes: | | |

**Outcomes**

*Copy and paste table for each outcome. (7 outcomes as per max recommended by Cochrane - QoL, anxiety, stress, depression, length of labour, pain management in labour, mode of birth)*

**Outcome 1**

|  | Description as stated in report/paper | | Location in text or source *(pg & /fig/table/other)* |
| --- | --- | --- | --- |
| Outcome name |  | |  |
| Time points measured *(specify whether from start or end of intervention)* |  | |  |
| Time points reported |  | |  |
| Outcome definition *(with diagnostic criteria if relevant)* |  | |  |
| Person measuring/ reporting (administered, self-report) |  | |  |
| Unit of measurement *(if relevant)* |  | |  |
| Scales: upper and lower limits *(indicate whether high or low score is good)* |  | |  |
| Is outcome/tool validated? | □ □ □  Yes No Unclear |  |  |
| Imputation of missing data *(e.g. assumptions made for ITT analysis)* |  | |  |
| Assumed risk estimate *(e.g. baseline or population risk noted in Background)* |  | |  |
| Power *(e.g. power & sample size calculation, level of power achieved)* |  | |  |
| Notes: | | | |

**Limitation and mitigation strategy**

| **Strength** |  |  |
| --- | --- | --- |
| **Limitations** |  |  |
| **Strategies to overcome the limitations** |  |  |
| **Notes:** | | |

**Data and analysis**

*Copy and paste the appropriate table for each outcome, including additional tables for each time point and subgroup as required.*

***Dichotomous outcome***

|  | Description as stated in report/paper | | | | | Location in text or source *(pg & ¶/fig/table/other)* |
| --- | --- | --- | --- | --- | --- | --- |
| Comparison |  | | | | |  |
| Outcome |  | | | | |  |
| Subgroup |  | | | | |  |
| Time point *(specify from start or end of intervention)* |  | | | | |  |
| Results | Intervention | | | Comparison | |  |
|  | No. with event | Total in group | | No. with event | Total in group |  |
|  |  |  | |  |  |  |
| Any other results reported *(e.g. odds ratio, risk difference, CI or P value)* |  | | | | |  |
| No. missing participants |  | | |  | |  |
| Reasons missing |  | | |  | |  |
| No. participants moved from other group |  | | |  | |  |
| Reasons moved |  | | |  | |  |
| Unit of analysis *(by individuals, cluster/groups or body parts)* |  | | | | |  |
| Statistical methods used and appropriateness of these *(e.g. adjustment for correlation)* |  | | | | |  |
| Reanalysis required? *(specify, e.g. correlation adjustment)* | □ □ □  Yes No Unclear | |  | | |  |
| Reanalysis possible? | □ □ □  Yes No Unclear | |  | | |  |
| Reanalysed results |  | | | | |  |
| Notes: | | | | | | |

***For RCT/CCT***

***Continuous outcome***

|  | | Description as stated in report/paper | | | | | Location in text or source *(pg & ¶/fig/table/other)* | |
| --- | --- | --- | --- | --- | --- | --- | --- | --- |
| Comparison | |  | | | | |  | |
| Outcome | |  | | | | |  | |
| Subgroup | |  | | | | |  | |
| Time point *(specify from start or end of intervention)* | |  | | | | |  | |
| Post-intervention or change from baseline? | |  | | | | |  | |
| Results | Intervention | | | Comparison | | |  | |
|  | Mean | SD *(or other variance, specify)* | No. participants | Mean | SD *(or other variance, specify)* | No. participants |  |  |
|  |  |  |  |  |  |  |  |  |
| Any other results reported *(e.g. mean difference, CI, P value)* | |  | | | | |  | |
| No. missing participants | |  | |  | | |  |  |
| Reasons missing | |  | |  | | |  |  |
| No. participants moved from other group | |  | |  | | |  |  |
| Reasons moved | |  | |  | | |  |  |
| Unit of analysis  *(individuals, cluster/ groups or body parts)* | |  | | | | |  | |
| Statistical methods used and appropriateness of these *(e.g. adjustment for correlation)* | |  | | | | |  | |
| Reanalysis required? *(specify)* | | □ □ □  Yes No Unclear | |  | | |  | |
| Reanalysis possible? | | □ □ □  Yes No Unclear | |  | | |  | |
| Reanalysed results | |  | | | | |  | |
| Notes: | | | | | | | | |

***Continuous outcome***

|  | | Description as stated in report/paper | | | | | | Location in text or source *(pg & ¶/fig/table/other)* | |
| --- | --- | --- | --- | --- | --- | --- | --- | --- | --- |
| Comparison | |  | | | | | |  | |
| Outcome | |  | | | | | |  | |
| Subgroup | |  | | | | | |  | |
| Time point *(specify from start or end of intervention)* | |  | | | | | |  | |
| Post-intervention or change from baseline? | |  | | | | | |  | |
| Results | Intervention | | | | Comparison | | |  | |
|  | Mean | SD *(or other variance, specify)* | No. participants | | Mean | SD *(or other variance, specify)* | No. participants |  | |
|  |  |  |  | |  |  |  |  |  |
| Any other results reported *(e.g. mean difference, CI, P value)* | |  | | | | | |  | |
| No. missing participants | |  | | |  | | |  |  |
| Reasons missing | |  | | |  | | |  |  |
| No. participants moved from other group | |  | | |  | | |  |  |
| Reasons moved | |  | | |  | | |  |  |
| Unit of analysis  *(individuals, cluster/ groups or body parts)* | |  | | | | | |  | |
| Statistical methods used and appropriateness of these *(e.g. adjustment for correlation)* | |  | | | | | |  | |
| Reanalysis required? *(specify)* | | □ □ □  Yes No Unclear | |  | | | |  | |
| Reanalysis possible? | | □ □ □  Yes No Unclear | |  | | | |  | |
| Reanalysed results | |  | | | | | |  | |
| Notes: | | | | | | | | |  |

***Other outcome***

|  | Description as stated in report/paper | | | | | Location in text or source *(pg & ¶/fig/table/other)* |
| --- | --- | --- | --- | --- | --- | --- |
| Comparison |  | | | | |  |
| Outcome |  | | | | |  |
| Subgroup |  | | | | |  |
| Time point *(specify from start or end of intervention)* |  | | | | |  |
| No. participants | Intervention | | | Control | |  |
|  |  | | |  | |  |
| Results | Intervention result | SE (or other variance) | | Control result | SE (or other variance) |  |
|  |  |  | |  |  |  |
|  | Overall results | | | SE (or other variance) | |  |
|  |  | | |  | |  |
| Any other results reported |  | | | | |  |
| No. missing participants |  | | |  | |  |
| Reasons missing |  | | |  | |  |
| No. participants moved from other group |  | | |  | |  |
| Reasons moved |  | | |  | |  |
| Unit of analysis *(by individuals, cluster/groups or body parts)* |  | | | | |  |
| Statistical methods used and appropriateness of these |  | | | | |  |
| Reanalysis required? *(specify)* | □ □ □  Yes No Unclear | |  | | |  |
| Reanalysis possible? | □ □ □  Yes No Unclear | |  | | |  |
| Reanalysed results |  | | | | |  |
| Notes: | | | | | | |

**Other information**

|  | **Description as stated in report/paper** | **Location in text or source** *(pg & ¶/fig/table/other)* |
| --- | --- | --- |
| **Key conclusions of study authors** |  |  |
| **References to other relevant studies** |  |  |
| **Correspondence required for further study information** *(from whom, what and when)* |  | |
| **Notes:** | | |
