# Supplementary material for: The characteristics and effectiveness of pregnancy yoga interventions: a systematic review and meta-analysis
Source: BMC Pregnancy Childbirth. 2022 Mar 25;22:250. doi: 10.1186/s12884-022-04474-9 (PMC8957136; doi:10.1186/s12884-022-04474-9)
Supplement: Supplementary file 4 — Additional file 4. Subgroup FITT principle of exercise prescription analysis. [file 12884_2022_4474_MOESM4_ESM.docx]

**Subgroup FITT principle of exercise prescription analysis**

***Frequency***

Eight studies reported that pregnant women were assigned to a moderate-frequency (three times weekly) yoga intervention [13, 29, 31, 32, 35, 37, 46, 50], sixteen studies assigned pregnant women to a low-frequency (once or twice weekly) yoga intervention [11, 34, 36, 39–42, 44, 45, 47–49, 52–55] and two studies assigned pregnant women to a high-frequency (daily) yoga intervention [30, 33]. Four studies did not report the frequency of the pregnancy yoga intervention [28, 38, 43, 51] and one study offered just a single session [8].

Subgroup analysis of the frequency of the yoga intervention across outcomes could not be conducted for perceived stress, anxiety, depression, duration of labour, pain management or quality of life. The test for subgroup differences for mode of birth suggest that there is a statistically significant subgroup effect for low-frequency yoga interventions of weekly or bi-weekly sessions (p<0.001) (Fig. 5a). There is low heterogeneity between the trials within the subgroup (I^2^=0%) suggesting the validity of the treatment effect estimate is reliable.

***Intensity***

Intensity was assessed based on taught yoga sessions as opposed to self-reported home practice. One study had just a single yoga session [8], eleven studies consisted of 6-12 yoga sessions [11, 34, 35, 41, 44, 45, 47, 52–55] and fifteen studies consisted of 12 sessions or more [13, 29–33, 36, 39, 40, 42, 46–50]. Four studies did not provide adequate information for analysis of intensity [28, 38, 43, 51].

Subgroup analysis looking at the intensity of the yoga interventions across outcomes was not possible for duration of labour, pain management or quality of life. The test for subgroup differences for perceived stress revealed that there is statistically significant subgroup effect for interventions with more than 12 sessions (p<0.001) (Fig. 5b). There is low heterogeneity between the trials within this subgroup (I^2^=0%) meaning the validity of the treatment effect estimate is reliable. The analysis for anxiety revealed that 6-12 sessions had a more significant impact on anxiety (p<0.001) than more than 12 sessions (Fig. 5c). There was substantial heterogeneity between the trials within this subgroup (I^2^=61%), therefore the validity of the treatment effect estimate is uncertain. For depression, the analysis demonstrated no statistically significant difference for interventions with 6-12 sessions (p=0.09) or more than 12 sessions (p=0.16) (Fig. 5d). An analysis of mode of birth showed that interventions with more than 12 sessions had a more significant positive impact on the rate of normal vaginal births (p=0.003) (Fig. 5e). There was substantial heterogeneity between the trials within this subgroup (I^2^=80%) meaning the validity of the treatment effect estimate is unreliable.

**Time**

Seven studies used short-time duration (≦ 45min) yoga sessions [34, 37, 39–42, 45], eleven studies used moderate-time duration (>45-≦60min) yoga sessions [8, 30–33, 35, 46, 47, 50, 53, 54] and nine studies used a long-duration session time (>60min) [11, 13, 29, 43, 44, 48, 49, 52, 55]. Four studies did not report the duration of the yoga sessions [28, 36, 38, 51].

Subgroup analysis looking at the duration of the yoga intervention across outcomes could not be carried out for duration of labour, mode of birth, pain management and quality of life. The test for subgroup differences for perceived stress demonstrated that long-duration yoga interventions greater than 60 minutes had a greater significant positive impact (p<0.001) than moderate-duration yoga interventions greater than 45 minutes but less than 60 minutes (Fig. 5f). There was low heterogeneity between the trials within this subgroup (I^2^=0%) meaning the validity of the treatment effect estimate is reliable. The same subgroup analysis for anxiety revealed that long-duration yoga interventions had a greater significant positive impact (p=0.007) (Fig. 5g). There was substantial heterogeneity between the trials within this subgroup (I^2^=82%) meaning the validity of the treatment effect estimate is unreliable. For depression, subgroup analysis demonstrated no significant difference in impact on depression scores between short (p=0.15), moderate (p=0.35) and long duration yoga interventions (p=0.27) (Fig. 5h).

**Type**

Eighteen studies reported the intervention consisted of a yoga session [8, 11, 33, 36, 39, 43–55], nine studies yoga therapy/integrated yoga therapy [13, 28–32, 34, 35, 37] and three studies yoga postures [40–42]. In addition, three studies [43, 48, 55] named the type of yoga as Hatha and one study as Ashtanga Vinyasa [44].

Subgroup analysis of the type of the yoga intervention provided across outcomes was not feasible for perceived stress, duration of labour, pain management and quality of life. The test for subgroup differences for anxiety suggest that there is a statistically significant subgroup effect for yoga sessions (p<0.001) and yoga therapy (p<0.001) compared to yoga postures (p=0.48) (Fig. 5i). The analysis for depression indicates a statistically significant subgroup effect for yoga therapy (p<0.001) but not for yoga postures (p=0.55) and yoga sessions (p=0.13) (Fig. 5j). The test for subgroup difference for mode of birth revealed that there is a statistically significant subgroup effect for yoga sessions (p<0.001) (Fig. 5k). There is substantial heterogeneity between the trials within the yoga sessions subgroup (I^2^=67%) meaning the validity of the treatment effect estimate is unreliable.
